# Corn peptides attenuate non-alcoholic fatty liver disease via PINK1/Parkin-mediated mitochondrial autophagy

**DOI:** 10.29219/fnr.v67.9547

**Published:** 2023-09-29

**Authors:** Zhicui Yao, Xiaoling Li, Wentao Wang, Peng Ren, Shiming Song, Haiyue Wang, Ying Xie, Xingbo Li, Zengning Li

**Affiliations:** 1 Department of Nutrition, Hebei Province Key Laboratory of Nutrition and Health, The First Hospital of Hebei Medical University, Shijiazhuang, Hebei Province, China; 2 College of Nursing, Hebei University of Chinese Medicine, Shijiazhuang, Hebei Province, China; 3 School of Nursing, Hebei Medical University, Shijiazhuang, Hebei Province, China

**Keywords:** corn peptides, mitochondrial autophagy, non-alcoholic fatty liver disease, PINK1/Parkin pathway

## Abstract

**Background:**

Corn peptides, a novel food prepared from corn gluten meal (CGM) by enzymatic hydrolysis or microbial fermentation, have attracted considerable interest owing to their various bioactive properties. However, the underlying mechanism of corn peptides attenuate non-alcoholic fatty liver disease (NAFLD) remains unclear.

**Objective:**

This study aimed to investigate the effect of corn peptides in NAFLD and to decipher the underlying mechanisms.

**Design:**

NAFLD was induced by a high-fat diet (HFD) for 10 weeks. Corn peptides were administered during the period. Human hepatocellular carcinomas (HepG2) cells induced by free fatty acids were used for this mechanism study.

**Results:**

Corn peptides alleviated HFD-induced histopathological changes, disorders of lipid metabolism, and mitochondrial damage. Moreover, corn peptides blocked mitophagy suppression by HFD based on the increased LC3, ATG7 expressions, as well as decreased P62 levels. Corn peptides increased the expression of proteins involved in fatty acid β-oxidation, such as PPARα and PGC-1α. Corn peptides also improved the Ser/Thr kinase PINK1 (PINK1) and the E3 ubiquitin ligase Parkin (Parkin) translocation to mitochondria, which is confirmed by immunofluorescence. Furthermore, stable knockdown of PINK1 by PINK1 SiRNA in HepG2 inhibited PINK1-Parkin-associated mitophagy and resulted in lipid accumulation.

**Conclusion:**

Corn peptides improved cell injury and ameliorated mitochondrial dysfunction and lipid accumulation via PINK1/Parkin-mediated autophagy in NAFLD. Thus, corn peptides could be a promising nutritional molecule with natural functions for preventing NAFLD.

## Popular scientific summary

- This study showed that corn peptides improved mitochondrial dysfunction of NAFLD.
- This study elucidated that corn peptides significantly ameliorated oxidative stress in NAFLD.
- This study demonstrated that corn peptides improved lipid accumulation of NAFLD via PINK1/Parkin-mediated mitochondrial autophagy in vivo and in vitro.

With rapid lifestyle transitions, the increasing burden of non-alcoholic fatty liver disease (NAFLD) in the world has emerged as a major public health issue (1, 2). Notably, the number of NAFLD patients increased from 18 to 29% within a decade in China (2, 3). The prevalence rates are rising more than twice as fast as in western countries. Predictively, the prevalence of NAFLD in China will reach 314.58 million cases by 2030, the highest increase in the world (4). Moreover, China has the youngest median age of NAFLD in the world, which implies that the greatest impact of its advanced complications will happen in the later decades.

NAFLD, a progressive disease, includes a spectrum of pathologies, ranging from simple hepatic steatosis to non-alcoholic steato-hepatitis (NASH), which may lead to liver fibrosis, cirrhosis, hepatocellular carcinoma (HCC), and death (5). As a complex disease, multiple factors could underlie the pathophysiology of NAFLD. However, convincing evidences have suggested that mitochondrial dysfunction may be a key pathogenesis involved in NAFLD (6). Mitochondrial dysfunction can cause energy depletion of hepatocytes and result in dysfunction of proton pump, increased permeability of mitochondrial inner membrane, rapid dissipation of membrane potential, mitochondrial swelling, increased reactive oxygen specie (ROS), mitochondrial DNA mutation, and ultimately hepatocyte inflammation. In conclusion, the disorder of liver lipid metabolism and its induced mitochondrial dysfunction in liver cells are key factors in the pathogenesis of NAFLD and the progression of liver inflammation.

Mitochondrial autophagy, a specialized form of autophagy, is activated to renew the damaged mitochondria and misfolded proteins during periods of stress to regulate cell death and maintain the homeostasis of lipid metabolism in the liver. The Ser/Thr kinase PINK1 (PINK1) and the E3 ubiquitin ligase Parkin (Parkin) are the two main regulators of mitophagy in mammalian cells. It has been well established that the PINK1/Parkin pathway is one of the classical pathways of mitochondrial autophagy (7). Parkin, as an ‘enhancer’ of mitochondrial autophagy signal, mediates the amplification of autophagy signal through further ubiquitination of mitochondrial proteins. The increased lipid accumulation in liver can decrease the level of autophagy affecting the role of autophagy in lipid degradation. Therefore, maintaining mitochondrial autophagy in hepatocytes can promote the degradation of excess lipid deposition, which is conducive to reducing the damage of lipid toxicity, and endoplasmic reticulum (ER) stress to hepatocytes is an important link in regulating the balance of hepatobiliary lipid metabolism. At present, effective drugs for the prevention or treatment of NAFLD are still lacking, and that drugs are mainly detoxified and metabolized in the liver. Inappropriate or excessive drug intake can increase hepatotoxicity, which leads to further damage the liver. So it is important to exploit food-borne liver protection functional food that does not exacerbate liver impairment or dysfunction for treating NAFLD to our country.

Corn peptides are a novel food prepared from corn gluten meal (CGM) by enzymatic hydrolysis or microbial fermentation and have low molecular weight with interesting structures and a high nutritional value. In addition, corn peptides are actively absorbed by the body and easy to digested, which can reduce the burden on the gastrointestinal tract improving gastrointestinal dysfunction and similar conditions. Recently, they have attracted attention due to their wide range of biological and pharmacological activities, which can help treat diseases with relatively few side effects. These bioactive properties include antioxidant activity (8), improvements in lipid and cholesterol (9), anti-obesity (10), and antihypertensive (11, 12). Corn peptides can accelerate alcohol metabolism and decrease serum aminotransferase activities to protect against alcohol-induced liver injury (13). In addition, corn peptides were also shown to exhibit a significantly hepatoprotective effect against some drug factors (14–16). However, the underlying mechanism of corn peptides protecting liver is still not very clear. Hence, further study will be necessary to elucidate the underlying mechanism of the hepatoprotective function of corn peptides and to explore whether it is involved in mitophagy regulation of NAFLD. This study aimed to determine the effect of corn peptides on hepatic steatosis both in vivo and in vitro and to further investigate whether they promoted hepatocyte autophagy by activating the PINK1–Parkin pathway to attenuate NAFLD.

## Materials and methods

### Animal experiments

#### Animal models

Six-week-old male Sprague–Dawley (SD) rats were purchased from Vital River Laboratory Animal Technology Co., Ltd. (Beijing, China; Certificate No.: SCXK2016-0006). The rats were housed in a specified living condition where temperature was maintained at 23 ± 1°C with a 12 h/12 h light/dark cycle and a relative humidity of 50%. Rats were acclimatized to laboratory conditions and fed a normal diet for a week before they were used in the experiments. The rats were provided food and drinking water ad libitum. Then, the rats were randomly divided into four groups with 10 rats in each group as follows: CON (control group, fed with normal diet); HFD (high-fat diet group, fed with a HFD [88% kcal from basal feed, 10% kcal from fat, 0.5% bile salt, and 1.5% cholesterol], purchased from Beijing Keao Xieli Feed Co., Ltd. (Beijing, China); CPs (800 mg/kg/d corn peptides + HFD); CCPs (control group + 800 mg/kg/d corn peptides). The special doses of corn peptides were calculated on the basis of the weekly average body weight of each group of rats and then were dissolved in distilled water and gavaged to each rat (2 mL). At the same time, the control group and the HFD model group were given 2 mL of distilled water by gavage. The entire experiment lasted for 10 weeks according to the result of preliminary experiment. During the experiment, the body weight, diet, and drinking water of the rats were recorded weekly.

Animal welfare and experimental procedures were performed strictly in accordance with the Guidelines of Rutgers University Institutional Animal Care and Use Committee (IACUC) and were approved by the Animal Experimentation Ethics Committee of First Hospital of Hebei Medical University (Permit No: 20200381).

### Body weight gain, liver weight, and liver index

At the end of the study, the rats were weighed and sacrificed under deep anesthesia following a 12 h overnight fast. After blood extraction, the liver was harvested after rinsed in phosphate-buffere dsalin (PBS) and weighted. The liver index was calculated as follows: liver index = (liver weight/body weight) × 100%. Part of the liver was fixed in formaldehyde at 4°C, while the remaining part was stored at -80°C for subsequent analyses after being quickly frozen in liquid nitrogen.

### Collection and biochemical analysis of serum and liver tissue

After 10 weeks of normal or HFD food intake, the serum was prepared by centrifugation at 4000r for 10 min at 4°C and then stored at -80°C for biochemical analyses.

Serum and liver tissue levels of triglycerides (TG), total cholesterol (TC), alanine aminotransferase (ALT), aspartate aminotransferase (AST) activities, low-density lipoprotein cholesterol (LDL-C), high-density lipoprotein cholesterol (HDL-C), as well as superoxide dismutase (SOD), malondialdehyde (MDA), and glutathione peroxidase (GSH-Px), were determined using a commercially available assay kit (Nanjing JianCheng Bioengineering Institute, Nanjing, China).

### Histopathological examination

Tissue sections (4 µm) embedded in paraffin were sectioned using amicrotome, deparaffinized, rehydrated, stained with hematoxylin & eosin (H&E), and then were observed under anoptical microscope (CX21, Olympus, Tokyo, Japan). Frozen liver sections were stained with Oil Red O (BSBA-4081, Beijing Zhongshan Jinqiao Biotechnology Co., Ltd., Beijing, China) to assess lipid accumulation under a microscope (Nikon FDX-35, Shanghai, China).

### Transmission electron microscopy analysis

Ultrastructure of mitochondria was observed using transmission electron microscope (TEM). Fresh liver fragments (1 mm^3^) were rapidly fixed in 2.5% glutaraldehyde (in phosphate buffer, pH 7.4) after harvested and then post-fixed in 1% osmium tetraoxide for 2 h in dark. Subsequently, the samples were dehydrated by an increasing concentrations gradient of alcohol and 100% acetone and embedded with EMBed 812 (SPI 90529-77-4, USA) overnight in 37°C. Ultrathin sections at 60–80 nm from Ultra microtome (Leica UC7, Germany) were stained by 2% uranium acetate and 2.6% lead citrate and then observed by TEM (HT7800, Japan).

### Cell culture and treatments

Human hepatocellular carcinomas (HepG2) cell line was purchased from the Chinese Academy of Sciences (Shanghai, China) and cultured in Dulbecco’s modified Eagle’s medium (DMEM, Hyclone, Logan, USA), supplemented with 10% fetal bovine serum (FBS), 1% penicillin/streptomyc, and at 37°C and 5% CO_2_. The induction medium contained free fatty acids (FFAs; palmitic and oleic acids at a molar ratio of 1:2 + 1% FFA-free bovine serum albumin (BSA, Sigma-Aldrich, St. Louis, MO, USA) at a final concentration of 1 mM for 24 h (17).

### Cell viability assay

HepG2 cells were seeded on a 96-well culture plate (1 × 10^4^/well) and 100 μL of DMEM medium containing different concentrations of corn peptides (0, 5, 10, and 15 mg/ml) for 24 h with or without FFAs. Three replicate wells were used. The cell vitality was determined using Cell Counting Kit-8 (CCK-8) (Solarbio, Beijing, China). 10 μL of CCK-8 solution was added to each well and then was incubated at 37°C for 1 h. The cell viability was evaluated by the optical density (OD) measured at 450 nm.

### Oil Red O staining and intracellular triglyceride (TG) assay

Oil Red O was used to stain in tracellular lipids. After removing the cell culture medium, the cells were fixed with 10% formaldehyde for 20 min, stained with Oil Red O for 15 min at room temperature, and then washed with distilled water. Cells were treated with 60% isopropanol for 10 min and then washed with distilled water. After counterstained with hematoxylin for 1 min, the cells were observed and imaged under the light microscope (Nikon FDX-35, Shanghai, China) and then the results were analyzed using the Image J software. Intracellular TG level was measured by the TG assay kit (Nanjing Jiancheng Bioengineering Institute, China), according to the manufacturer’s instructions.

### Measurement of mitochondrial inner membrane potential (MMP)

Changes in MMP were measured using the tetraethyl benzimida-zolyl carbocyanine iodide (JC-1) kit. HepG2 cells (2 × 10^5^/well) were seeded in 24-well plates, incubated for 24 h, and then treated with corn peptides (0, 5, 10, and 15 mg/ml) with or without FFAs for 24 h. After rinsing with pre-cooled PBS, the cells were incubated with JC-1 probe for 20 min at 37°C in the dark (18). Subsequently, cells were observed under a fluorescence microscope (magnification, 200×) (Nikon, Japan) immediately after washed with PBS to remove the free JC-1 probe. Red fluorescence was detected at 490 nm excitation and 530 nm emission, and green fluorescence was detected at 525 nm excitation and 590 nm emission. The ratio of red fluorescence and green fluorescence presents the changes in MMP.

### Measurement of ROS

Cellular ROS was measured using the (2’,7’ - Dichlorodihydrofluorescein diacetate) DCFH-DA. HepG2 cells were seeded in a 24-well plate (3 × 10^5^/well) and then incubated at 37°C for 24 h. The cells were treated with corn peptides (0, 5, 10, and 15 mg/ml) for 24 h with or without FFAs and then harvested. After washed with PBS, the cells were incubated with DCFH-DA dilution (10 µM, serum-free medium:DCFH-DA=1000:1), incubated at 37°C for 30 min in the dark, and then washed three times with serum-free medium to remove free ROS probe. Finally, the DCFH-DA fluorescence intensity was measured by fluorescence microscope (Nikon, Japan).

### Immunofluorescence

For immunofluorescence, the liver paraffin sections were deparaffinized and rehydrated, and then tissue antigen was repaired using the antigen retrieval solution and blocked with BSA for 30 min. They were then incubated with primary antibody in a wet box overnight followed by incubation with secondary antibodies for 50 min in dark. The primary antibodies for immunofluorescence staining were as follows: PINK1 (1:200, Affinity) and Parkin (1:500, Affinity). The nuclei were stained with (4’,6-diamidino-2-phenylindole) DAPI after washed three times with PBS. Finally, the tissue sections were observed under a fluorescence microscope (Nikon, Japan) after sealed using neutral resin sealing.

### Western blot analysis

The protein of liver tissues and HepG2 cells was lysed and extracted using Radio Immunoprecipitation Assay (RIPA) lysis buffer (Beijing Beyotime, China), and the concentration was quantified using a BCA assay kit (Solarbio, PC0020, Beijing, China). The denatured protein samples (50 µg) from each group were loaded on 10% SDS-polyacrylamide gel electrophoresis (SDS-PAGE) gel and transferred onto polyvinylidene fluoride membranes (PVDF) (Millipore, Bedford, MA, USA). Each PVDF membrane was blocked with 1×TBST containing 5% skimmed milk and incubated with primary antibodies at 4°C refrigerator overnight and incubated with secondary antibodies for 1 h in the dark. All the primary antibodies in the Western blot experiment are as follows: PGC1α (1:1000, Proteintech), PPARα (1:1000, Proteintech), PINK1 (1:1000, Proteintech), Parkin (1:1000, Affinity), ATG7 (1:1000, Affinity), SQSTM1/p62 (1:1000, Affinity), FAS (1:1000, ab128856), SREBP-1c (1:1000, A15586), LC3 I/II (1:1000, AB Clonal), and β-actin (1:10000, AB Clonal). The chemiluminescence intensity of all membranes was visualized with enhanced chemiluminescence system. The gray value of protein strips was analyzed using the Image J software.

### Statistical analysis

All data statistical analyses in this study were performed using the SPSS21 software (IBM, Chicago, IL, USA). Differences among the groups were identified by one-way ANOVA, followed by the LSD or Dunnett’s tests. All data are presented as mean ± standard deviation (SD). *P* < 0.05 was considered statistically significant. All experiments were repeated at least three times.

## Results

### Corn peptides prevent HFD-induced hepatic steatosis in rats

**Fig. 1:**
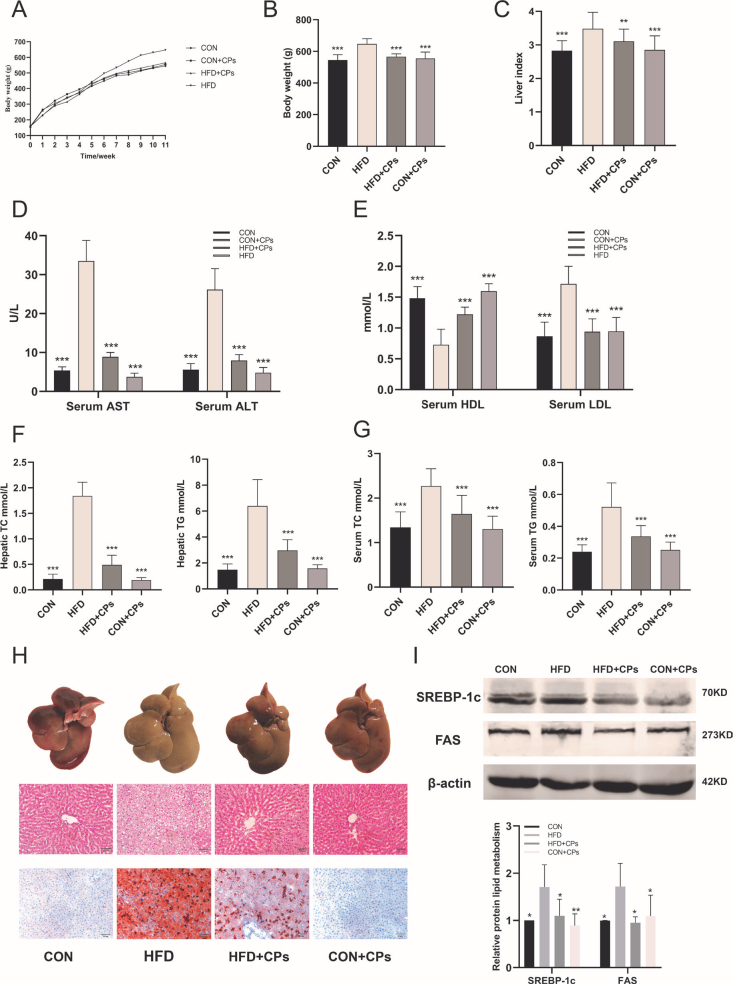
Corn peptides prevent HFD-induced hepatic steatosis in rats. (A) Body weight of SD male rats by week (*n* = 10); (B) body weight of SD male rats in 10 weeks (*n* = 10); (C) live index (*n* = 10). (D) Serum ALT and AST (*n* = 10); (E) serum LDL-C and HDL-C (*n* = 10); (F) hepatic TC and TG (*n* = 10); (G) serum TC and TG (*n* = 10); (H) the liver appearance; liver sections stained with H&E; liver sections stained with Oil Red O (magnification 200×); (I) the lipoprotein expression of SREBP-1c and FAS. Data are presented as mean ± SD (*n* = 5). Mean values with different letters are significantly different (^*^P < 0.05, ^**^P < 0.01, ^***^P <0.001 vs. HFD). HFD: high-fat diet; CPs: corn peptides.

To investigate the impact of corn peptides on obesity induced by HFD, the body weight of rats in each of group was monitored. The initial body weight of all six-week-old male rats was 158.75 ± 8.51g. The body weight gain of rats in the HFD model group was increased starting from 5 weeks (Fig. 1A). After 10 weeks of treatment, the body weight gains and the liver index in the HFD model group were significantly higher compared to the control group (Fig. 1B, C), accompanied by increasing TG, TC, and LDL-C and significantly reducing HDL-C concentrations both in liver tissue and serum. However, corn peptides treatment delayed these trends (Fig. 1D–G, *P* < 0.05). For gross morphology, enlarged volume and pale yellow appearance of livers were observed in the HFD model group, which were greatly alleviated by corn peptides treatment (Fig. 1H). In addition, to determine the histological effects of corn peptides on hepatic steatosis, liver sections were stained with H&E and Oil Red O to detect the injured and lipid accumulation. Histologic analysis revealed an extensive increase in lipid droplets in HFD model group rats (Fig. 1H), which was prevented considerably by corn peptides consistent with the TG level. Furthermore, corn peptides decreased the lipoprotein expression such as SREBP-1c and FAS (Fig. 1I). The aforementioned results indicated corn peptides could prevent HFD-induced hepatic steatosis in rats.

### Corn peptides ameliorated HFD-induced oxidative stress in rats

**Fig. 2:**
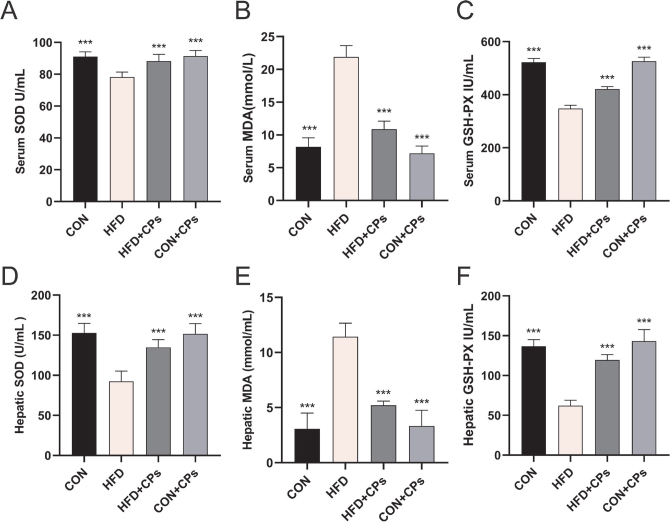
Corn peptides alleviated HFD-induced oxidative stress in rats. (A) Serum SOD level; (B) serum MDA level; (C) serum GSH-Px level; (D) hepatic SOD activity; (E) hepatic MDA level; (F) hepatic GSH-Px level. Data are presented as mean ± SD (*n* = 10). Mean values with different letters are significantly different (^*^P < 0.05, ^**^P < 0.01, ^***^P < 0.001 vs. HFD). HFD: high-fat diet; CPs: corn peptides; SOD: superoxide dismutase; MDA: malondialdehyde; GSH-Px: glutathione peroxidase.

To investigate the effect of corn peptides on oxidative stress, we measured the key antioxidant defense components in liver and blood serum, such as SOD, MDA, and GSH-Px. The administration of corn peptides significantly elevated the level of SOD and MDA and inhibited the superoxide accumulation of GSH-Px compared with the HFD model group (Fig. 2A–F).

### Corn peptides ameliorated HFD-induced mitochondria damage and promoted mitochondria autophagy in rats

**Fig. 3:**
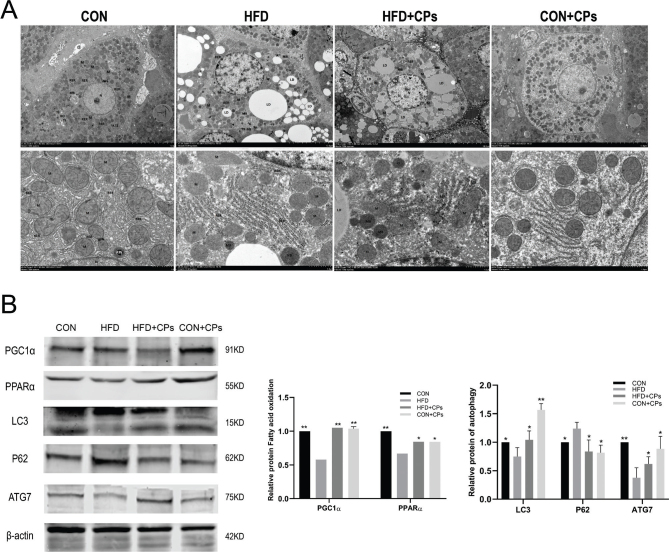
Corn peptides ameliorated HFD-induced mitochondrial damage and promoted mitochondrial autophagy in rats. (A) The ultrastructure of liver tissues electron transmission observed under the TEM. (B) The protein expression of PGC1α, PPARα, P62, ATG7, and LC3I/II. Data are presented as mean ± SD (*n* = 5). Mean values with different letters are significantly different (^*^P < 0.05, ^**^P < 0.01 vs. HFD).

To investigate the effect of corn peptides on the mitochondrial ultrastructure, TEM was used to directly assess the mitochondria ultrastructure in rat liver. As shown in the pictures of TEM, swelling, lacking cristae, and destruction of the inner membranes were observed in mitochondria in the HFD model group. In contrast, corn peptides treatment apparently ameliorated the abnormalities of mitochondria induced by HFD (Fig. 3A). Damaged or fragmented mitochondria can be removed through mitochondrial autophagy to control the quality of mitochondria and maintain mitochondrial homeostasis. To further detect the capacity of mitochondrial fatty acid oxidation and autophagy, the protein expression of PGC1α, PPARα, P62, ATG7, and LC3I/II was analyzed by Western blot. As anticipated, after 10 weeks of HFD, the fatty acid oxidation-related proteins expression such as PGC1α, PPARα, autophagy-related proteins, ATG7, and LC3I/II was notably declined, while P62 protein expression increased compared to the control group; corn peptides treatment reversed these changes (Fig. 3B). These results indicated that corn peptides ameliorated HFD-induced mitochondrial damage and promoted mitochondrial autophagy in rats.

### Corn peptides activated PINK1-Parkin signaling pathway in rats’ liver

**Fig. 4:**
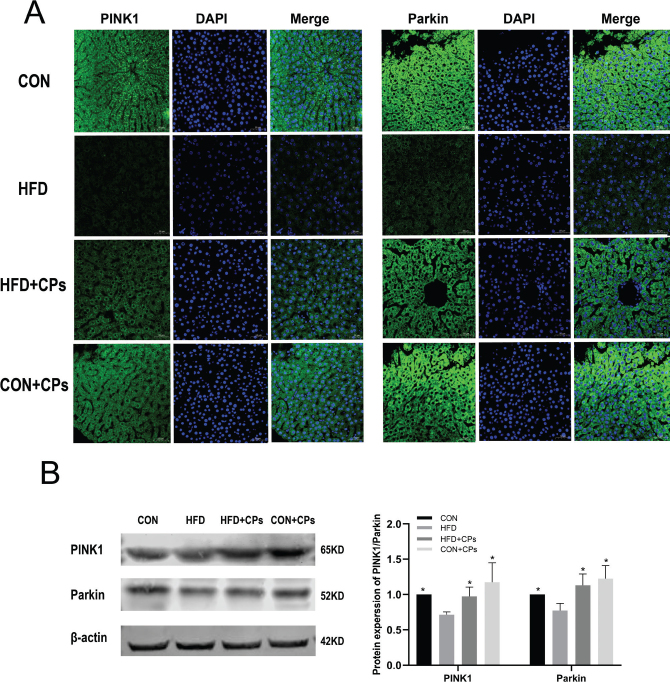
Corn peptides activated PINK1-Parkin signaling pathway in rats’ liver. (A) Immunofluorescence staining of PINK1 and Parkin. (B) The protein expression of PINK1 and Parkin. Data are presented as mean ± SD (*n* = 5). Mean values with different letters are significantly different (^*^P < 0.05 vs. HFD).

To explore the autophagy pathway regulated by corn peptides, PINK1 and Parkin, the two critical proteins in the PINK1/Parkin pathway were detected by Western blot and immunofluorescence. The liver of the HFD model group showed less amounts of mitochondria located by PINK1 and Parkin. However, corn peptides treatment significantly enhanced the expression of PINK1 and Parkin (Fig. 4A and B).

### Corn peptides prevented FFAs-induced lipid accumulation in HepG2 cells

**Fig. 5:**
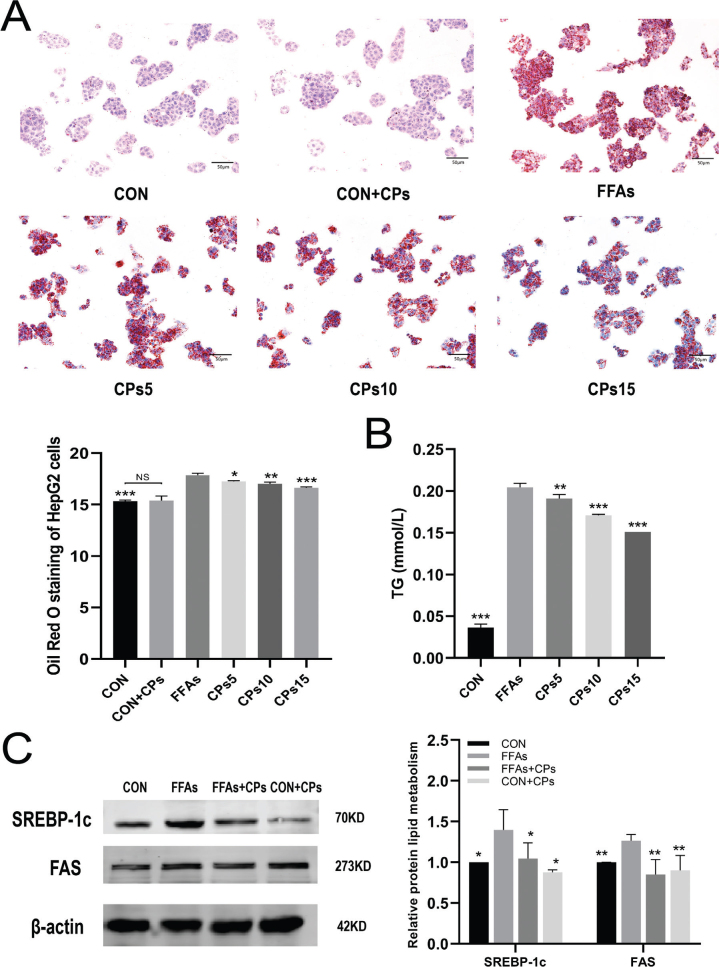
Corn peptides prevented FFAs-induced lipid accumulation in HepG2 cells. (A) Oil Red O staining in HepG2 cells (magnification 200×). (B) The intracellular TG content in HepG2 cells. (C) The protein expression of SREBP-1c and FAS. Results were presented as mean ± SD. ^*^P < 0.05, ^**^P < 0.01, ^***^P < 0.001 versus FFAs group (*n* = 3). FFAs: free fatty acids; CPs5: 5 mg/ml corn peptides in induction media; CPs10: 10 mg/ml corn peptides in induction media; CPs15: 15 mg/ml corn peptides in induction media; CCPs: 15 mg/ml corn peptides in control media. The experiment was repeated at least three times.

To ascertain the effect of corn peptides on lipid accumulation in FFAs-induced HepG2 cells, we measured TG levels intracellular and stained with Oil Red O. Results showed that lipid droplets increased in HepG2 cells induced by FFAs (Fig. 5A and B). In addition, the high-dose group of corn peptides decreased the lipoprotein expression such as SREBP-1c and FAS induced by FFAs induced in HepG2 cells (Fig. 5C).

### Corn peptides alleviated FFAs-induced oxidative stress in HepG2 cells

**Fig. 6:**
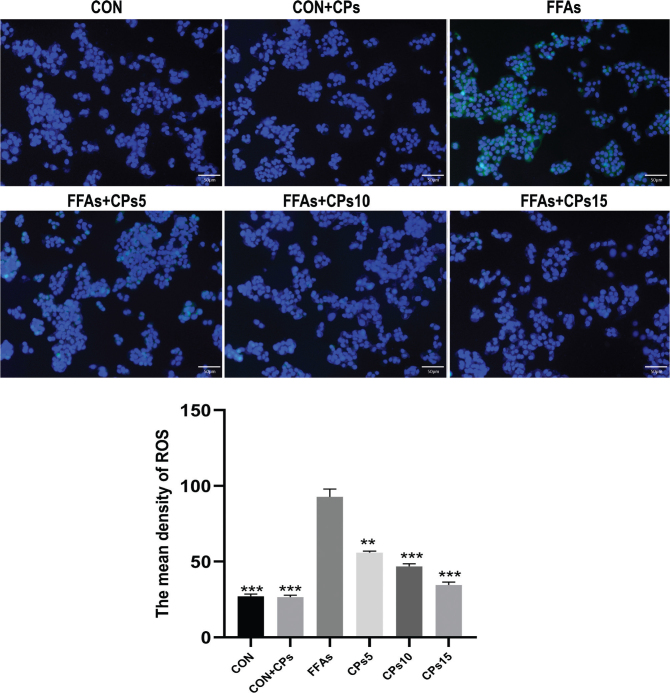
Corn peptides decreased FFAs-induced ROS in HepG2 cells. Cells were treated with different corn peptides concentrations for 24 h, and ROS levels were detected by immunofluorescence (magnification, ×200). Results were presented as mean ± SD. ^**^P < 0.01, ^***^P < 0.001 versus FFAs group (*n* = 3). FFAs: free fatty acids; CPs5: 5 mg/ml corn peptides in induction media; CPs10: 10 mg/ml corn peptides in induction media; CPs15: 15 mg/ml corn peptides in induction media; CCPs: 15 mg/ml corn peptides in control media. The experiment was repeated at least three times.

The effect of corn peptides on ROS generation in HepG2 cells was measured using DCFH-DA fluorescent probe. As expected, FFAs aggregation resulted in a significant increase in cellular ROS levels. Corn peptides treatment significantly decreased the fluorescence intensity of DCF in a dose-dependent manner (Fig. 6).

### Corn peptides ameliorated FFAs-induced mitochondrial damage and promoted mitochondrial autophagy in HepG2 cells

**Fig. 7:**
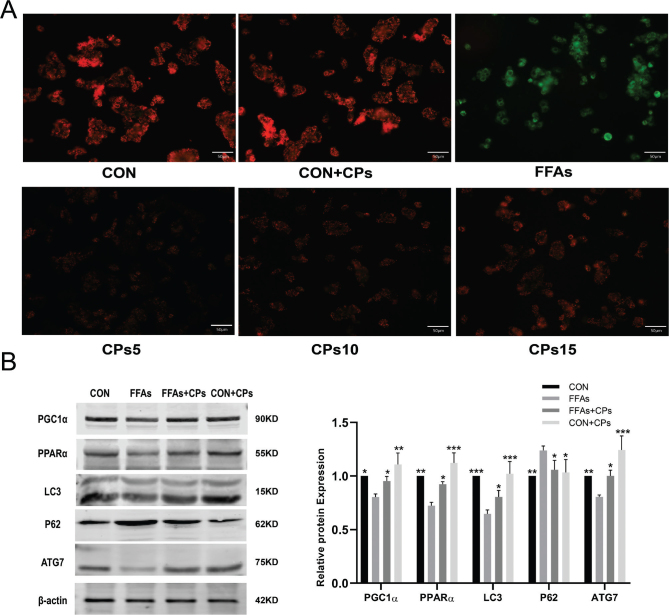
Corn peptides ameliorated mitochondrial damage and promoted mitochondrial autophagy in HepG2 cells. (A) Changes in the MMP were assessed by detecting the red fluorescence and green fluorescence ratio (magnification, ×200). (B) The protein expression of PGC1α, PPARα, ATG7, LC3I/II, and P62. Results were presented as mean ± SD. *P < 0.05, **P < 0.01, ***P < 0.001 versus FFAs group (*n* = 3). FFAs: free fatty acids; CPs5: 5 mg/ml corn peptides in induction media; CPs10: 10 mg/ml corn peptides in induction media; CPs15: 15 mg/ml corn peptides in induction media; CCPs: 15 mg/ml corn peptides in control media. The experiment was repeated at least three times.

In order to verify the effect of corn peptides on mitochondrial membrane potential, the JC-1 fluorescent needle was used in HepG2 cells. The expression of green fluorescence in the cells treated with corn peptides was significantly increased than that of the FFAs-induced group (Fig. 7A). This detection result confirmed that corn peptides treatment ameliorated mitochondrial damage. Western blot results showed that the high-dose group of corn peptides significantly upregulated the autophagy-related protein expressions, such as ATG7, LC3I/II, and decreased P62 level, and the fatty acid β-oxidation protein expression of PGC1α and PPARα was increased (Fig. 7B).

### Corn peptides promoted mitochondrial autophagy in HepG2 cells via activating PINK1-Parkin signaling pathway

**Fig. 8:**
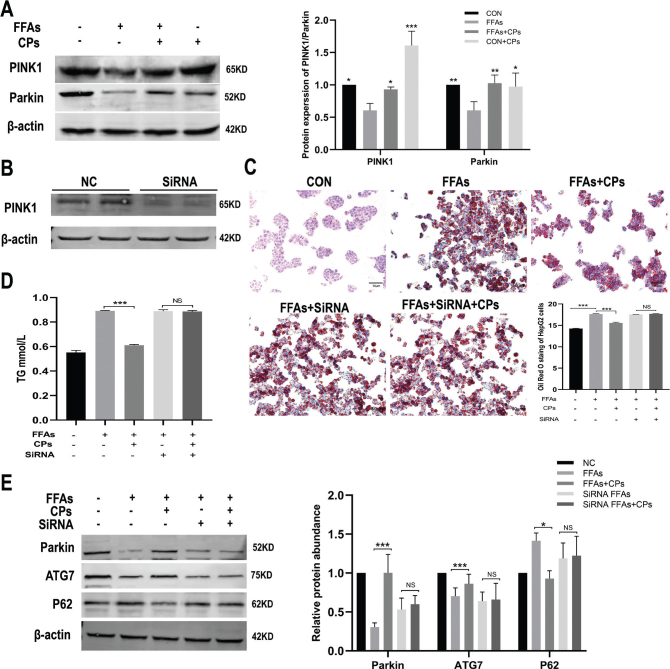
Corn peptides promoted mitochondrial autophagy in HepG2 cells via activating PINK1-Parkin signaling pathway. (A) The protein levels of PINK1 and Parkin in FFAs-induced HepG2 cells. (B) The protein levels of PINK1 in HepG2 cells transferred by siRNA. (C) Oil Red O stain in HepG2 cells transferred by siRNA (magnification, ×200). (D) The intracellular TG content in HepG2 cells transferred by siRNA. (E) The protein levels of Parkin, P62, and ATG7 in HepG2 cells transferred by siRNA. Results were presented as mean ± SD. ^*^P < 0.05, ^**^P < 0.01, ^***^P < 0.001 versus FFAs group (*n* = 3). FFAs: free fatty acids; CPs: 15 mg/ml corn peptides in control media. The experiment was repeated at least three times.

To verify whether corn peptides promoted mitochondrial autophagy in HepG2 cells via activating PINK1-Parkin signaling pathway, the protein expression of PINK1 and Parkin was analyzed by Western blot in HepG2 cells with or without siRNA. The results showed that the expression of PINK1 and Parkin showed a trend of gradually decreased in FFAs-induced HepG2 cells, which were reversed by corn peptides (Fig. 8A). Furthermore, Western blotting clearly showed that PINK1 protein was suppressed in HepG2 cells transfected with PINK1 siRNA (Fig. 8B). We next tested whether stable knockdown of PINK1 would affect the lipid metabolism and PINK1/Parkin-dependent mitophagy of HepG2 cells. As expected, corn peptides had no significant effect on lipid accumulation evaluated by Oil Red O stain and intracellular TG content after PINK1 was knocked out in FFAs-induced HepG2 cells (Fig. 8C and D). Western blotting analysis showed that Parkin protein and mitochondrial autophagy-related proteins P62 and ATG7 were also not increased by corn peptides after PINK1 knockdown (Fig. 8C). These data indicated that corn peptides promoted mitochondrial autophagy in HepG2 cells partly via activating PINK1-Parkin signaling pathway.

## Discussion

Foodborne corn peptides are typically small sequence of 2–20 amino acids with molecular masses between 300 and 1,000 Da. Increasing evidence (19–22) suggests that it has exhibited multiple biological activities, including anti-obesity, antihypertension, hypolipidemic, antioxidant, metal-binding activities, the ability to accelerate alcohol metabolism, and resultantly hepatoprotective effects. The most beneficial function of corn peptides is of antioxidant activity that it could scavenge reactive oxygen species directly (23), which has been studied through various chemical assays (24). Results in this study showed that corn peptides could effectively remove ROS in HepG2 cell induced by FFAs and increase antioxidant enzymes such as SOD, MDA, and GSH-Px in HFD rats. The result is consistent with Li et al. (25). A previous study showed that corn peptides were high rich in antioxidant residues, such as Glu, Leu, Ala, Pro, Phe, and Tyr, which could be the reason for the antioxidant effect.

Hepatic lipid metabolism disorder and its induced mitochondrial dysfunction are key factors in the pathogenesis of NAFLD and the progression of liver inflammation (26, 27). Mitochondrial autophagy can degrade damaged mitochondria and misfolded proteins in liver to regulate cell death and is an important pathway to regulate lipid metabolism and eliminate damaged mitochondria in liver (28). Lipid toxicity and ER stress are prone to occur if the autophagy of hepatocytes is inhibited. Therefore, maintaining mitochondrial autophagy of hepatocytes can promote the degradation of excess lipid deposition and regulate the balance of liver lipid metabolism.

The process of autophagosome formation mainly involves three main steps: initiation of complex with uncoordinated UNC-51-like kinase 1 (ULK1), nucleation of complex with Beclin-I–III phosphatidylinositol 3-kinase (PI3K), and lipidation of microtubule-associated protein 1 light chain 3 (LC3). LC3 is an important marker of autophagy activation, and its expression can reflect the degree of autophagy (29). In this study, corn peptides supplementation greatly increased the protein level of LC3II/I and decreased the P62 expression meanwhile. The result of TEM showed that the number of autophagosomes increased significantly, and the number and shape of lipid droplets decreased significantly in the group of corn peptides administration. Similar results were confirmed in FFAs-induced HepG2 cell. These results indicated that corn peptides improved morphology and function of mitochondrial and promoted autophagy. Furthermore, we also found corn peptides rescued Parkin mitochondrial translocation inducing mitophagy in hepatocytes in HFD mice and FFA-induced cells. The occurrence of mitochondrial autophagy is a result of multifactor regulation, which is a process co-mediated by multi-cytokine and channel protein. There are many molecular pathways involved in the mediation (30), among which, the PINK1/Parkin pathway is the most mature autophagy signaling pathway in eukaryotic cells at present. PINK1/Parkin-pathway-mediated mitochondrial autophagy was first discovered in the nervous system and is one of the pathological mechanisms of Parkinson’s disease (31–33). At the same time, PINK1 can enhance the antagonism of mitochondria against toxic substances (staphylosporin and rotenone), realize the self-cleaning renewal of mitochondria, and play a key role in mitochondria-related diseases (34). Recent studies (35–37) have found that PINK1/Parkin-mediated mitochondrial autophagy is also involved in the occurrence and development of liver-related diseases, including NAFLD, liver fibrosis, HCC, etc. In this study, it is observed that PINK1 and Parkin protein levels were decreased both in the liver of HFD rats and FFAs-induced HepG2 cells, which were reversed by corn peptides administration. To further dissect the role of corn peptides in the PINK1-Parkin-mediated mitophagy regulating the lipid metabolism, we knocked down the expression of PINK1 using siRNA and found that the absence of PINK1 abolished the protective effects of corn peptides against lipid accumulation and prevented the promotion of autophagy, suggesting that the corn peptides-induced autophagy of HepG2 cells was partly due to the activation of the PINK1/Parkin pathway.

In summary, understanding the complex therapeutic mechanisms of corn peptides in NAFLD may be an important step in improving therapeutic outcomes. This study demonstrated that corn peptides improved cell injury and ameliorated mitochondrial dysfunction and lipid accumulation via PINK1/Parkin-mediated autophagy in NAFLD. Thus, the PINK1/Parkin autophagy pathway may represent a novel therapeutic target of corn peptides for NAFLD. Corn peptides could be a promising nutritional molecule with natural functions for preventing NAFLD. In light of these results, we are conducting clinical validation in human NAFLD patients to further clarify the effect and mechanism of corn peptides in improving NAFLD.

## Conflict of interest and funding

The authors declare that they have no known competing financial interests or personal relationships that could have appeared to influence the work reported in this paper. This work was financially supported by the grants from the National Natural Science Foundation of China (82073531).

## Authors’ contributions

Zengning Li designed and coordinated the study, Zhicui Yao performed the experiments and wrote the manuscript, Wentao Wang and Peng Ren performed the experiments and acquired and analyzed the data; Xiao-Ling Li, Hai-Yue Wang, Ying Xie, and Xingbo Li reviewed the manuscript; all authors approved the final version of the article.

## Ethics statement

This study was approved by the Animal Ethics Committee of the First Hospital of Hebei Medical University (Permit No. 20200381).

## Data availability statement

The datasets used and analyzed during this study are available from the corresponding author upon reasonable request.
